# Dual inhibition of BRD4 and PI3K-AKT by SF2523 suppresses human renal cell carcinoma cell growth

**DOI:** 10.18632/oncotarget.21432

**Published:** 2017-09-30

**Authors:** Hua Zhu, Jia-Hui Mao, Yin Wang, Dong-Hua Gu, Xiao-Dong Pan, Yuxi Shan, Bing Zheng

**Affiliations:** ^1^ The Department of Urology, The Second Affiliated Hospital of Soochow University, Suzhou, China; ^2^ The Department of Urology, The Second Affiliated Hospital of Nantong University, Nantong, China; ^3^ Department of Pathophysiology, Nantong University School of Medicine, Nantong, China; ^4^ Institute of Neuroscience, Soochow University, Suzhou, China

**Keywords:** renal cell carcinoma (RCC), BRD4, PI3K-AKT-mTOR, SF2523, molecule-targeted therapy

## Abstract

Bromodomain-containing protein 4 (BRD4) and PI3K-AKT are both important for renal cell carcinoma (RCC) development and progression. SF2523 is a BRD4 and PI3K-AKT dual inhibitor. The present study demonstrated that SF2523 was cytotoxic and anti-proliferative to established RCC cell lines (786-O and A498) and primary human RCC cells. SF2523 induced activation of caspase and apoptosis in RCC cells. Further, SF2523 disrupted RCC cell cycle progression and inhibited cell migration *in vitro*. At the signaling level, SF2523 in-activated PI3K-AKT-mTOR, and downregulated BRD4-dependent proteins, Bcl-2 and Myc, in RCC cells. Remarkably, SF2523 was more efficient than Wortmannin (the PI3K inhibitor) and JQ1 (the BRD4 specific inhibitor) in killing RCC cells. *In vivo*, SF2523 administration at well-tolerated doses suppressed 786-O xenograft tumor growth in severe combined immunodeficient (SCID) mice. Together, our results suggest that concurrent blockage of BRD4 and PI3K-AKT signalings by SF2523 efficiently inhibits RCC cell growth *in vitro* and *in vivo*.

## INTRODUCTION

Total nephroureterectomy of early-stage and defined Renal cell carcinoma (RCC) tumor is only curable way clinically [1–4]. Yet, RCC is typically diagnosed at advanced-stages with local invasion and/or systematic metastasis. The prognosis of these RCC patients is often extremely poor [3, 5–8]. Molecule-targeted therapy has received broad attentions for RCC field [9]. Over the past years, our group is dedicated to indentifying novel oncotarget proteins for RCC, which shall help to develop possible intervention strategies [10–14].

Bromodomain-containing protein 4 (BRD4) is a well-studied member of the human BET (bromodomain and extraterminal) family proteins [15–17]. During the process of mitosis, BRD4 binds directly to acetylated-histones, which is required for maintaining chromatin structure in the daughter cells [15–17]. Additionally, BRD4 is also crucial for transcription machinery's association with the specific chromatin regions to ensure the early re-initiation of transcription after mitosis [15–17]. When facing transcription pause, BRD4 is shown to recruit P-TEFb (the positive transcription elongation factor b) and phosphorylate the RNA Pol II, allowing transcription elongation [15–17].

Recent studies have recognized BRD4 as a novel oncotarget protein, possibly due to its effect in the regulation of cell cycle progression [18–22]. BRD4 is over-expressed in multiple cancers. It is required for the expression of several key oncogenes, including Myc and Bcl-2 [18–22]. BRD4 knockdown, on the other hand, inhibited cancer cell growth, and decreased G1 phase cells [18–22]. JQ1, the small molecule BRD4 inhibitor, is shown to competitively displace BRD4 from acetylated-histones [18, 20–23]. Inhibition of BRD4 by JQ1 was shown to potently inhibit human cancer cell growth [18, 20–23].

Recent studies have developed SF2523 [24, 25] as a dual inhibitor of BRD4 and PI3K (phosphatidylinositol 3-kinase)-AKT, the latter is another key oncogenic pathway that is vital for RCC initiation and progression [26, 27]. The present research studied the potential effect of this duel inhibitor against RCC cells *in vitro* and *in vivo*.

## RESULTS

### SF2523 was cytotoxic and anti-proliferative to RCC cells

**Figure 1:**
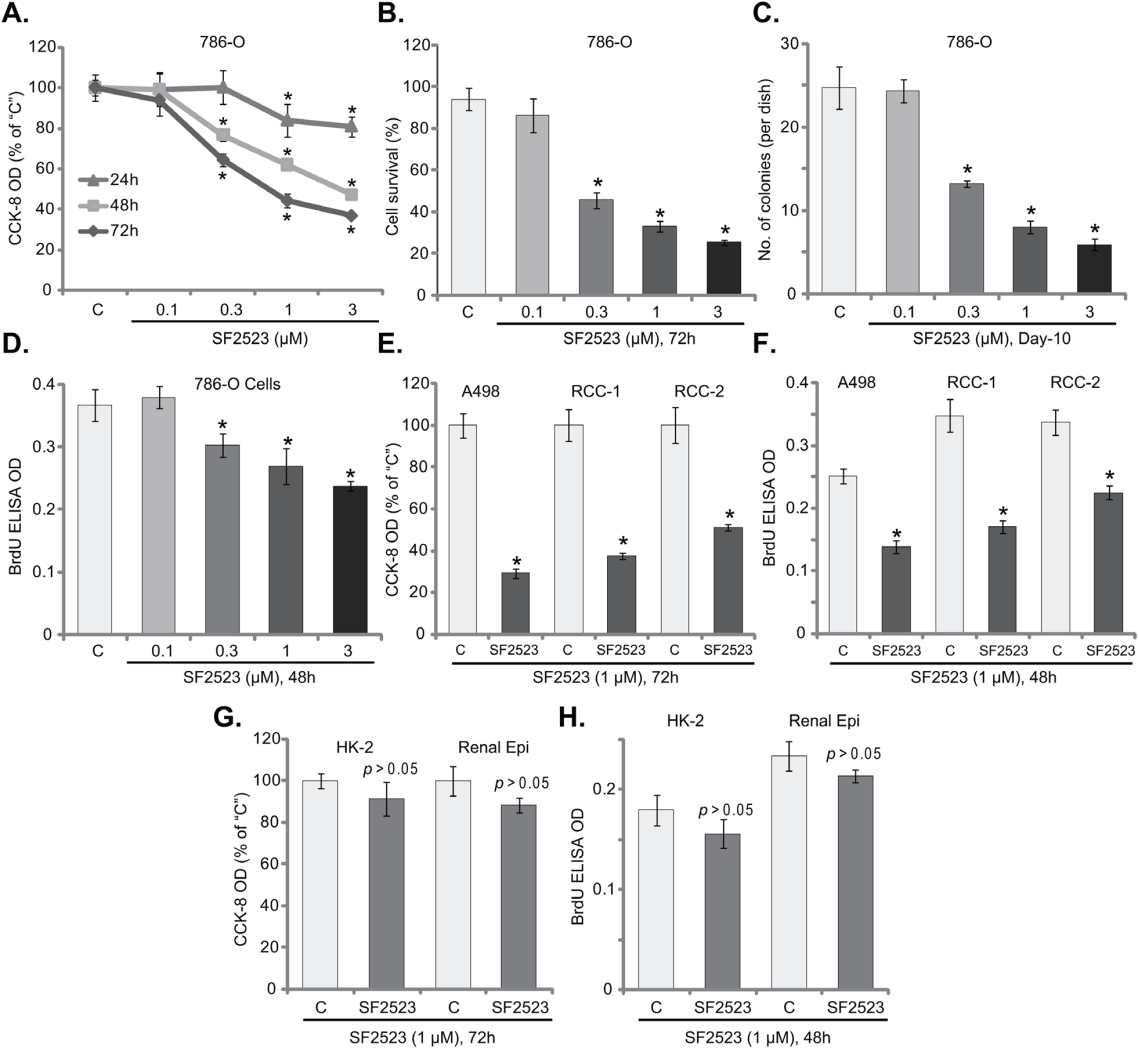
SF2523 was cytotoxic and anti-proliferative to RCC cells Established human RCC cell lines (786-O and A498), the primary human RCC cells (“RCC-1/2” lines), HK-2 tubular epithelial cells as well as the primary human renal epithelial cells (“Renal Epi”) were treated with indicated concentration of SF2523 for the applied time; Cell survival was tested by the listed assays mentioned in the text (**A-C, E** and **G**); Cell proliferation was assessed by the BrdU ELISA assay (**D, F** and **H**). Data were expressed as mean ± standard deviation (SD, n=5). The data in each figure were summarizing one set of experiment. ^*^p < 0.05 vs. untreated control group (“C”). Vehicle control (0.1% of DMSO) failed to change survival and proliferation of tested cells. Experiments in this figure were repeated four times, and similar results were obtained.

In order to study the potential effect of SF2523 on RCC cells, the established 786-O RCC cells [10, 11, 14] were maintained in FBS-containing complete medium and were treated with SF2523 at different concentrations. CCK-8 assay was employed to test cell viability. As displayed in Figure 1A, treatment with SF2523 indeed inhibited 786-O cell survival. Notably, SF2523-induced anti-survival activity was time-dependent (Figure 1A). Meanwhile, a dose-dependent response by SF2523 was also noticed (Figure 1A). SF2523, at 0.1 μM, was ineffective to inhibit 786-O cell survival (Figure 1A). The IC-50 of SF2523 (at 72 hours) was around 1 μM (Figure 1A). Trypan blue staining assay results further demonstrated that SF2523 (at 0.3-3.0 μM, 72 hours) decreased the number of viable (Trypan blue negative) 786-O cells (Figure 1B). Additionally, the clonogenicity assay results found that the number of viable 786-O colonies was decreased sharply after SF2523 (0.3-3.0 μM, renewed every two days) treatment (Figure 1C). The proliferation of 786-O cells was also tested by examining BrdU incorporation. The results in Figure 1D demonstrated that SF2523 treatment in 786-O cells dose-dependently inhibited BrdU ELISA OD, suggesting proliferation inhibition. SF2523, at 0.1 μM, was again ineffective (Figure 1D).

Next, we analyzed the potential activity of SF2523 in other RCC cells. As described in our previous studies [10, 11, 14], two lines of primary human RCC cells, namely “RCC-1/2”, as well as the other established RCC cell line, A498, were treated with SF2523 (at 1 μM). CCK-8 assay results in Figure 1E confirmed that SF2523 was anti-survival/cytotoxic to both A498 cells and two lines of the primary RCC cells. The CCK-8 OD was reduced significantly following SF2523 (1 μM, 72 hours) treatment in the RCC cells (Figure 1E). Furthermore, proliferation of the RCC cells, again tested by the BrdU ELISA assay, was also inhibited by SF2523 (1 μM, 48 hours) (Figure 1F). These results suggest that SF2523 was cytotoxic and anti-proliferative to both established and primary human RCC cells.

The potential effect of SF2523 to non-cancerous renal cells was also tested. HK-2 tubule epithelial cells and the primary human renal epithelial cells were cultured (see the previous study [13]) and treated with SF2523 (1 μM). Intriguingly, CCK-8 assay results in Figure 1G showed that SF2523 treatment (1 μM, 72 hours) was non-cytotoxic to the renal epithelial cells. The CCK-8 OD was almost unchanged before and after SF2523 treatment (Figure 1G). Meanwhile, the BrdU incorporation was also not significantly changed by SF2523 treatment (1 μM, 48 hours) in epithelial cells (Figure 1H). These results indicate that SF2523 was uniquely non-cytotoxic to normal renal epithelial cells.

### SF2523 induces profound apoptosis activation in RCC cells

**Figure 2:**
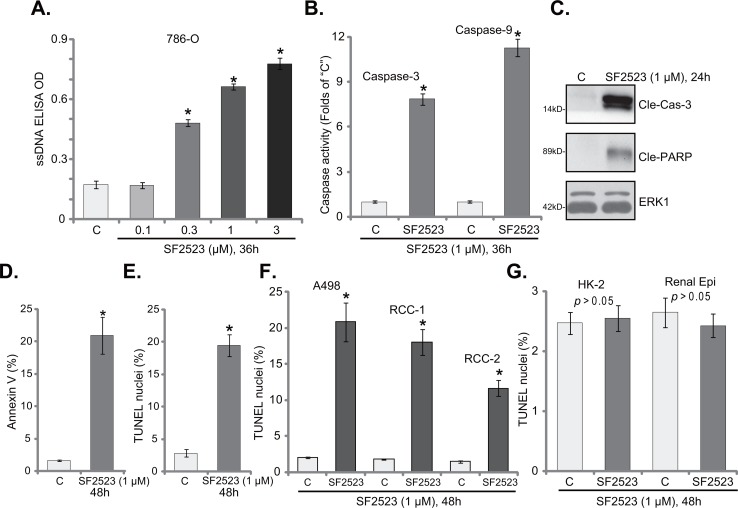
SF2523 provokes RCC cell apoptosis Established human RCC cell lines (786-O and A498), the primary human RCC cells (“RCC-1/2” lines), HK-2 tubular epithelial cells as well as the primary human renal epithelial cells (“Renal Epi”) were treated with indicated concentration of SF2523 for the applied time; Cell apoptosis was tested by the assays mentioned in the text **(A, B, D-G)**; Expressions of cleaved-caspase-3 (“Cle-Cas-3”) and cleaved-PARP (Cle-PARP) were also tested, with ERK1 as the loading control (**C**, for 786-O cells). Data were expressed as mean ± standard deviation (SD, n=5). The data in this figure were summarizing one set of experiment. ^*^p < 0.05 vs. untreated control group (“C”). Vehicle control (0.1% of DMSO) failed to change apoptosis of the tested cells. Experiments in this figure were repeated three times, and similar results were obtained.

Apoptosis induction is a major reason of cancer cell growth inhibition/cell death [28–32]. A number of anti-cancer agents provoke cell apoptosis to kill cancer cells [29–31, 33, 34]. Over-production of single strand DNA (ssDNA) is often detected as the indicator of cell apoptosis. Here we show that SF2523 treatment dose-dependently increased ssDNA content in 786-O RCC cells (Figure 2A). Further, SF2523 (1 μM) increased activities of caspase-3 and caspase-9 in 786-O cells (Figure 2B). Meanwhile, cleavages of caspase-3 and PARP (poly (ADP-ribose) polymerase) were observed in SF2523 (1 μM)-treated cells (Figure 2C). Additionally, SF2523 (1 μM) significantly increased the number of Annexin V-labeled (Figure 2D) and TUNEL-stained (Figure 2E) 786-O cells.

TUNEL assay was also employed to test the potential activity of SF2523 on other RCC cells. Results in Figure 1F clearly showed that SF2523 (1 μM, 48 hours) treatment significantly increased the number of TUNEL staining in A498 cells and in two lines of the primary human RCC cells. Thus, SF2523 is also pro-apoptotic in these RCC cells. On the other hand, the very same SF2523 treatment (1 μM, 48 hours) in HK-2 cells and the primary human renal epithelial cells was unable to induce significant apoptosis (TUNEL assay, Figure 1G), again showing a selective response of this compound only to the cancer cells.

### SF2523 disrupts RCC cell cycle progression and inhibits cell migration

**Figure 3:**
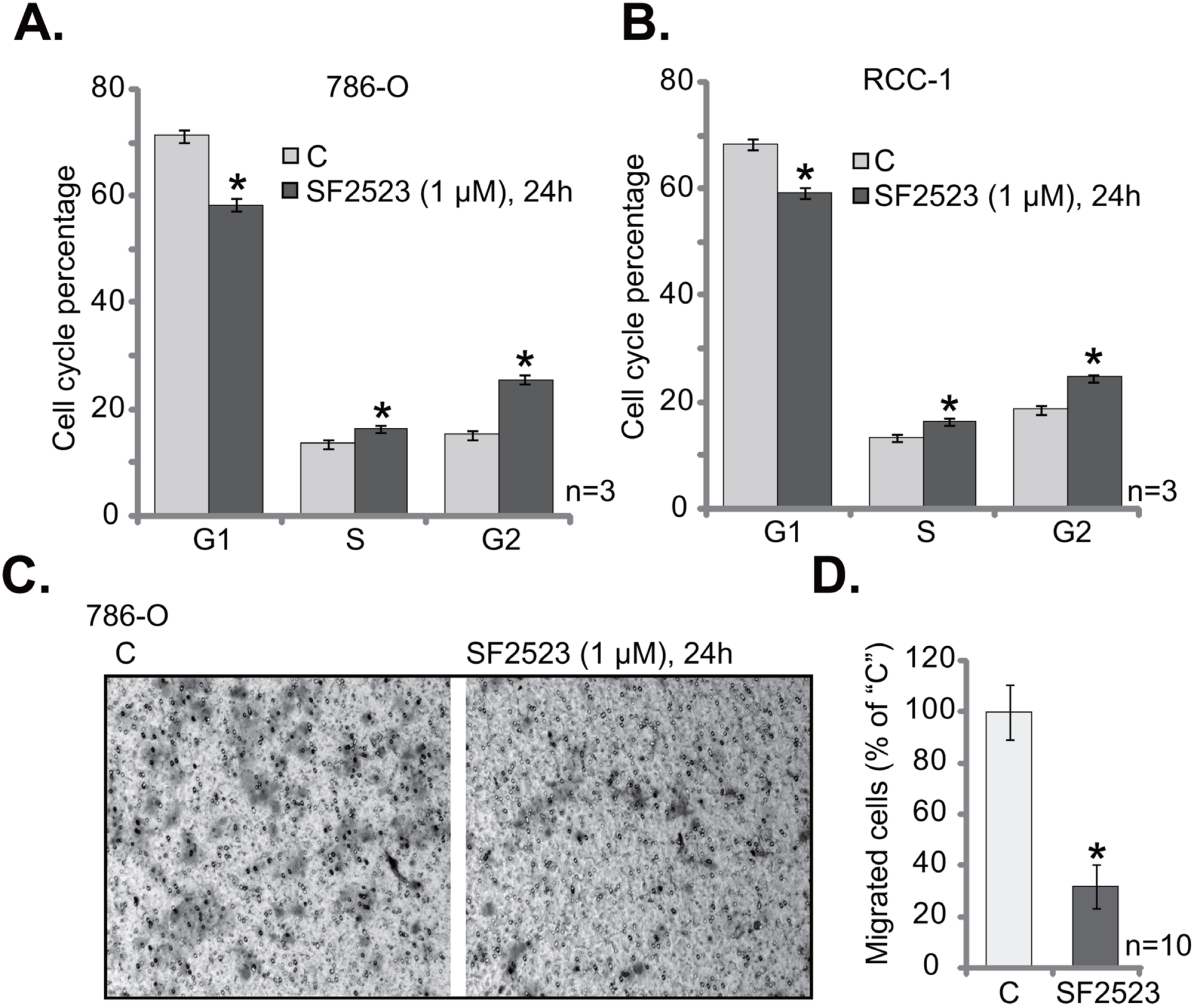
SF2523 disrupts RCC cell cycle progression and inhibits cell migration 786-O cells or the primary human RCC cells (“RCC-1” line) were treated with/out 1 μM of SF2523 for 24 hours, cell cycle distribution (**A** and **B**) and cell migration (**C** and **D**) were tested by the assays mentioned in the text. Data were expressed as mean ± standard deviation (SD). The data in this figure were summarizing one set of experiment. ^*^p < 0.05 vs. untreated control group (“C”). Experiments in this figure were repeated three times, and similar results were obtained.

The PI3K-AKT and BRD4 signaling pathways are both important for cell cycle progression and cell migration. The propidium iodide/fluorescence-activated cell sorter (PI-FACS) assay results in Figure 3A showed that SF2523 treatment (1 μM, 24 hours) in 786-O cells significantly decreased G1 phase percentage, but increased G2- and S-phase cell percentages. Similarly in the primary human RCC cells (“RCC 1” line), SF2523 induced G1 phase decrease and S-G2 phase increase (Figure 3B). Next, the “Transwell” assay was performed to test the cell migration. Representative Transwell images showed that SF2523 treatment (1 μM, 24 hours) significantly inhibited the number of “migrated” 786-O cells (on the bottom of the Transwell) (Figure 3C). Quantified results integrating 10 random “Transwell” views of each condition further confirmed that SF2523 inhibited 786-O cell migration *in vitro* (Figure 3D). These results show that SF2523 disrupts RCC cell cycle progression and inhibits cell migration.

### SF2523 blocks PI3K-AKT-mTOR and BRD4 signalings in RCC cells

**Figure 4:**
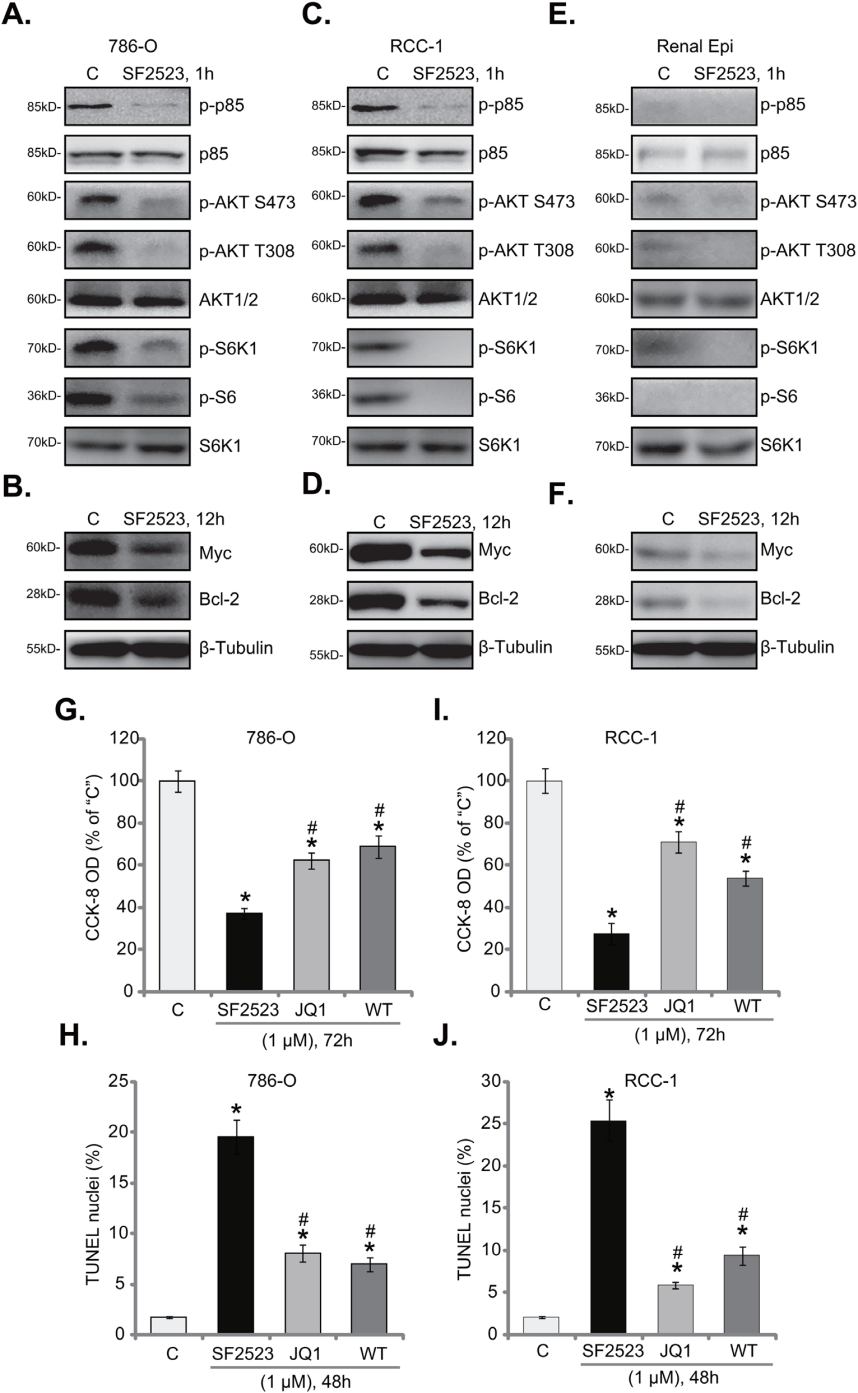
SF2523 blocks PI3K-AKT-mTOR and BRD4 signalings in RCC cells Western blotting analysis of lysates from listed cells treated with/out SF2523 (1 μM), cell lysates were probed with specified antibodies **(A-F)**. 786-O cells or the primary human RCC cells (“RCC-1” line) were treated with/out 1 μM of SF2523, Wortmannin (“WT”) or JQ1; Cell survival was tested by CCK-8 assay (**G** and **I**); Cell apoptosis was assessed by the TUNEL staining assay (**H** and **J**). Data were expressed as mean ± standard deviation (SD). The data in this figure were summarizing one set of experiment. ^*^p < 0.05 vs. untreated control group (“C”). ^#^ *p* < 0.05 vs. SF2523 group. Experiments in this figure were repeated four times, and similar results were obtained.

SF2523 is a novel PI3K-BRD4 duel inhibitor [24, 25]. We next tested the two signaling cascades in RCC cells with SF2523 treatment. As shown in Figure 4A, SF2523 (1 μM, 1 hour) significantly inhibited p85 phosphorylation in 786-O cells, suggesting PI3K inhibition. Consequently, activation of PI3K downstream signalings, AKT and mTOR complex 1 (mTORC1), was also largely inhibited (Figure 4A). Activation of AKT was tested by phosphorylations of AKT at both Ser-473 and Thr-308 [35], mTORC1 activation was reflected by phosphorylations of its substrates, S6K1 and S6 [36] (Figure 4A). Expressions of total kinases were unchanged before and after SF2523 treatment (Figure 4A). Further studies showed that expressions of BRD4-dependent proteins, including Bcl-2 and Myc [18, 21], were also dramatically downregulated in SF2523 (1 μM, 12 hours)-treated cells (Figure 4B).

These findings suggest that SF2523 simultaneously blocked PI3K-AKT-mTORC1 and BRD4 signalings in 786-O cells. Very similar results were also obtained in the primary human RCC cells. Western blotting assay results in primary “RCC-1” cells confirmed that PI3K-AKT-mTORC1 activation (Figure 4C) and BRD4-dependent proteins (Bcl-2/Myc, Figure 4D) were both significantly downregulated following SF2523 (1 μM) treatment. Importantly, in the primary renal epithelial cells, basal (“untreated”) PI3K-AKT-mTORC1 activation (Figure 4E) and Bcl-2/Myc expression (Figure 4F) were relatively low, this could explain why SF2523 treatment was in-effective to the non-cancerous epithelial cells.

We next compared SF2523's activity with the pan PI3K-AKT-mTOR inhibitor Wortmannin [37] and the BRD4 specific inhibitor JQ1 [18, 20]. In 786-O cells, SF2523 (1 μM) was significantly more potent than the same concentration of Wortmannin and JQ1 in inhibiting cell survival (Figure 4G) and inducing cell apoptosis (Figure 4H). Very similar results were also observed in the primary RCC cells, where SF2523 was more efficient (than Wortmannin or JQ1) in killing cells (Figure 4I and 4J). Thus, concurrent blockage of PI3K-BRD4 signalings is apparently more efficient than inhibition of each single pathway in killing RCC cells.

### SF2523 inhibits 786-O xenograft tumor growth *in vivo*

**Figure 5:**
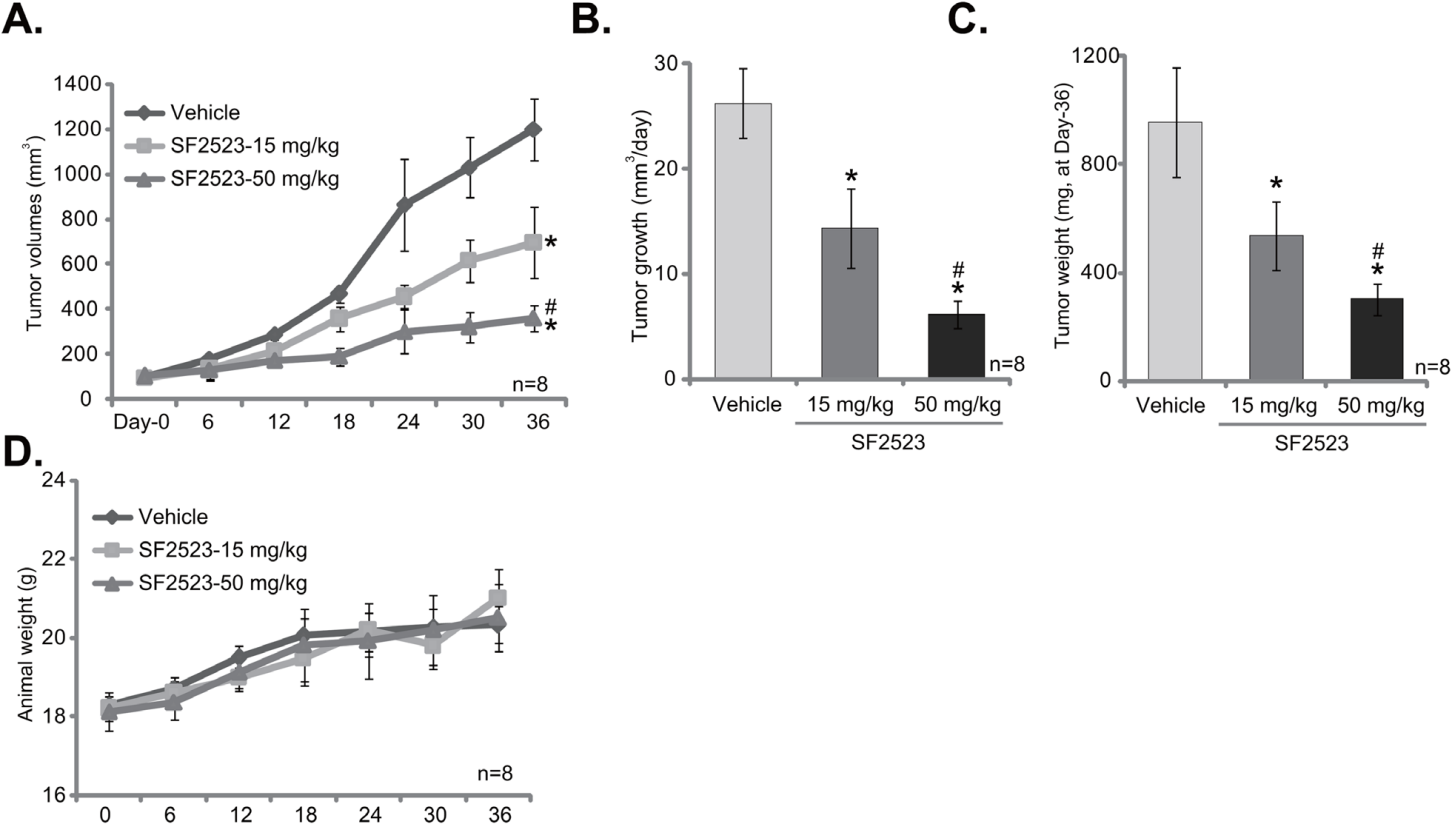
SF2523 inhibits 786-O xenograft tumor growth *in vivo* 786-O cells were implanted in SCID mice. When tumors reached 100 mm^3^ (within three weeks), animals were randomly divided into three groups. One group was treated with vehicle control (Saline) and the other two with SF2523 (15 and 50 mg/kg, every other day). The number of mice per experimental group, n = 8. Estimated tumor volume **(A)** and daily tumor growth **(B)** were presented. At the end of experiment (Day-36), tumors of the each group were isolated and weighted **(C)**. SF2523 showed no gross toxicity to mice, as there was no notable change in body weight **(D)**. ^*^p < 0.05 vs. “Vehicle group”. ^#^ *p* < 0.05 vs. SF2523 at 15 mg/kg group.

The potential anti-tumor activity of SF2523 *in vivo* was tested. As previously described [11, 13], 786-O cells were injected *s.c.* to the SCID nude mice. Within three weeks, the xenograft tumor model was established, and tumors were all around 100 mm^3^ in volume (Labeled as “Day-0”). Tumor-bearing mice were then randomly assigned into three groups, receiving SF2523 (based on the previous treatment regimens [24]) or vehicle treatment. Tumor growth curve results in Figure 5A display that administration of SF2523 (15/50 mg/kg body weight, every other day) significantly inhibited 786-O tumor growth in SCID mice. Estimated daily tumor growth, in mm^3^ per day [38], was also decreased with SF2523 administration (Figure 5B). At the end of experiments (“Day-36”), tumors of each group were isolated and weighted. As demonstrated, tumors of SF2523 treatment groups were significantly lighter than vehicle control tumors (Figure 5C). Notably, SF2523 displayed a dose-dependent response in inhibiting 786-O tumor growth *in vivo* (Figure 5A-5C). SF2523 at 50mg/kg was significantly more potent than 15 mg/kg in suppressing 786-O tumors (Figure 5A-5C). In line with the previous findings [24], administration of SF2523 at tested doses didn't change the animals’ body weight (Figure 5D). We also failed to detect any significant toxicities in the tested mice.

## DISCUSSION

PI3K-AKT-mTOR cascade is arguably one of the most important oncogenic signaling [39–42]. Our studies [11–13] and others [26, 43, 44] have demonstrated that PI3K-AKT-mTOR signaling is often hyper-activated in RCC, which positively contributes to multiple key cancerous behaviors, including uncontrolled cell survival, proliferation, migration, as well as apoptosis resistance, cancer metastasis and angiogenesis [26, 43, 44]. Therefore, PI3K-AKT-mTOR pathway is a pivotal oncotarget for RCC treatment. PI3K-AKT-mTOR inhibitors have been developed and tested in preclinical and clinical cancer studies [40–42]. In the current study, we show that SF2523 blocked PI3K-AKT-mTOR signaling activation in RCC cells. Importantly, the anti-RCC cell activity by SF2523 was significantly more potent the pan PI3K-AKT-mTOR inhibitor Wortmannin. Therefore, concurrent inhibition of PI3K-AKT and BRD4 signalings should be a better strategy to inhibit RCC cells.

Tang's study has demonstrated that Myc pathway is often over-activated in RCC, which is essential for cancer cell growth and apoptosis resistance [45]. Furge *et al.*, have provided computational and genetic evidences to support that Myc over-activation is associated with RCC aggression and progression [46]. Intriguingly, hypoxia-inducible factor 1α (HIF1α) is shown to inhibit RCC by suppressing Myc activity [47]. Bcl-2 is a well-established anti-apoptosis protein [48], its expression is often increased in RCC, which is associated with resistance to chemotherapeutic agents radiation therapy [49]. It has been extensively studied that BRD4 is crucial for the expressions of both Myc and Bcl-2 [18, 20–22, 24]. In the current study, we show that BRD4 inhibition by SF2523 induced dramatic downregulation of Myc and Bcl-2 in RCC cells. This should also explain its superior activity against RCC cells.

Concurrent activation of multiple oncogenic signaling pathways has become a hallmark of RCC and many other cancers [50]. These signalings, working together or separately, cause cancer cell progression [50]. Often, inhibition of one single pathway will only result in minor to moderate anti-tumor activity. In the current study, we show that dual inhibition of PI3K-AKT and BRD4 signalings by SF2523 potently inhibited RCC cell growth *in vitro* and *in vivo*. Its activity was more potent than inhibition of each single pathway.

## MATERIALS AND METHODS

### Reagents, chemicals and antibodies

SF2523, JQ1 and Wortmannin were purchased from Selleck (Shanghai, China). All the antibodies of the present study were purchased from Santa Cruz Biotechnology (Santa Cruz, CA) and Cell Signaling Technologies (Beverly, MA). Reagents for cell culture were provided by Gibco (Suzhou, China).

### Culture of cell lines

Established human RCC cell lines (786-O and A489) as well as HK-2 tubule epithelial cells were described previously [10–14].

### Primary culture of human RCC cells

As described [12], the tissue specimens were obtained from two written-informed RCC patients (Patient 1, male, 54-years old; Patient 2, male, 43-years old) (see our previous study [14]) with total nephroureterectomy. The two patients were both administrated at the Second Affiliated Hospital of Nantong University (Nantong, China), and received no treatment prior to the surgery. The RCC tumor tissues and surrounding normal renal epithelial tissues were separated very carefully under the microscopy. Tissues were digested using collagenase I (Sigma, 0.05% w/v) incubation. Individual cells were pelleted, rinsed and filtered. A total of two lines of primary human RCC cells, namely “RCC-1 and RCC-2”, as well as one line of primary human renal epithelial cells were established in the present study. Primary human cells were cultured in the described medium [12–14]. Experiments and protocols were approved by the Ethics Review Board (ERB) of Soochow University (Suzhou, China). Experiments were according to the principles of Declaration of Helsinki.

### CCK-8 assay

Cell Counting Kit-8 (CCK-8; Dojindo, Kumamoto, Japan) was applied to detect cell survival. The optical density (OD) of CCK-8 was measured at 450 nm.

### Trypan blue staining assay

The cells negative of Trypan blue staining was labeled as the viable cells. Following the applied treatment, the survival cell percentage was determined by an automatic cell counter (Roche, Shanghai, China).

### Clonogenicity assay

The detailed protocol of clonogenicity assay was described in our previous studies [10–14]. Briefly, 786-O RCC cells were incubated with SF2523-containing medium every 2 days for a total of 10 days. Afterwards, the number of viable 786-O colonies of each dish were counted manually.

### BrdU ELISA assay of cell growth

As described [13, 14], the BrdU ELISA kit (Cell Signaling Tech, Shanghai, China) was utilized to assess cell growth *in vitro*.

### Annexin V assay of cell apoptosis

As reported, cells with the described SF2523 treatment were further stained with Annexin V-FITC (5 μg/mL) and propidium iodide (PI, 5 μg/mL) (Invitrogen, Shanghai, China), which were then sorted via fluorescence-activated cell sorting (FACS) machine from Becton-Dickinson machine (San Jose, CA). Annexin V-stained cells were labeled as the apoptotic cells.

### Caspase-3/-9 activity assay

As described [11], 10 μg of cytosolic extracts per treatment were incubated with the caspase assay buffer [11] along with the caspase-3/-9 substrate [11] for 2 hours at room temperature. AFC (7-amido-4-(trifluoromethyl)-coumarin) release was quantified via the Fluoroskan system [11].

### ELISA assay of ssDNA

An ELISA kit testing cellular ssDNA (Roche, Shanghai, China) was employed to test ssDNA fragmentation from 30 μg cellular lysate proteins (per condition). The detailed protocol was attached to the kit. ELISA OD at 405 nM was recorded.

### TUNEL assay

Following the applied SF2523 treatment, cells were subjected to the TUNEL dye assay, using the protocol attached (Biyuntian, Wuxi, China). At least 300 cells of five random views (1 : 100, light microphage) of each condition were analyzed to calculate TUNEL ratio.

### Transwell assay

The 8.0-μm-pore Transwell plate (Fisher, Shanghai, China) was utilized to test RCC cell migration *in vitro*. Briefly, 786-O cells were plated at 1× 10^5^/mL in each well, and 1 mL of complete medium (with/out SF2523) was added to the bottom of the plate. Cells were allowed to migrate for 24 hours. The non-migrated 786-O cells were removed. Then, the migrated cells on the bottom surface were fixed and stained. After air-drying, the stained (migrated) cells were counted. Cells in at least 10 random views under each condition were counted.

### Western blotting assay

As described [10–14], the cell lysates were fractionated on the 10-12% SDS-page gels, and were transferred to PVDF membranes. The blots were probed with designated primary antibodies and corresponding second antibodies (Santa Cruz Biotech). Enhanced chemiluminescence (ECL) reagents (GE Healthcare, Shanghai, China) were applied to visualize the interested bands under the x-ray film development. Band intensity was quantified using the ImageJ software.

### Xenograft model

The female severe combined immunodeficient (SCID) mice of 4-5 weeks old (17-18g weight) were purchased from Nantong University Animal Laboratories (Nantong, China). 786-O cells (5 × 10^6^ per mouse) were injected *s.c.* to the flanks of the mice. Within three week, the tumor xenografts were established with the volume around 100 mm^3^ of each tumor. Mice (n=8 each group) were treated as described. Mice body weight and bi-dimensional tumor measurements were taken every 6 days. Tumor volume was calculated as described [11]. The animal protocol was approved by the Institutional Animal Care and Use Committee (IACUC) and Ethics Review Board (ERB) of Soochow University.

### Statistical analysis

Data were expressed as mean ± standard deviation (SD). The data in each figure were summarizing one set of experiment. Statistical analyses were performed by one-way analysis of variance (ANOVA) with the GraphPad software. IC-50 was calculated by the SPSS 17.0 software. Significance was set at *p* < 0.05.

## CONCLUSION

Together, we show that concurrent blockage of PI3K-AKT and BRD4 signalings by SF2523 efficiently inhibits human RCC cell growth *in vitro* and *in vivo*.
